# Fall risk after total hip replacement: A functional correlation study

**DOI:** 10.6026/973206300214706

**Published:** 2025-12-15

**Authors:** Manoj Agnihotri, Ajit Dabholkar

**Affiliations:** 1 Department of Musculoskeletal Physiotherapy, Babandada Patil College of Physiotherapy, Taloja, Navi Mumbai, India; 2 Department of Sports Physiotherapy, School of Physiotherapy, D.Y. Patil Deemed to be University, Nerul, Navi Mumbai, Maharashtra, India

**Keywords:** Total hip arthroplasty (THA), falls, harris hip score, sit-to-stand, alternate step test, timed up and go, geriatric rehabilitation

## Abstract

Falls are a common issue among older adults after total hip arthroplasty (THA), with factors such as reduced mobility and fear of falling contributing. Therefore, it is of interest to examine recovery and fall-related concerns in patients following total hip arthroplasty (THA). It used measures such as the Harris Hip Score (HHS) and the Falls Efficacy Scale (FES) to assess progress. The study found that the Sit-to-Stand test (STS) was strongly linked to fall confidence, emphasising its value in monitoring recovery and fall risk. This study is the identification of key functional measures (such as the Sit-to-Stand test and Falls Efficacy Scale) in assessing recovery and fall risk in older adults post-total hip arthroplasty, emphasizing the role of physical and psychological factors in improving fall-related confidence and mobility.

## Background:

Falls remain a considerable challenge in the early stages of recovery among older adults following total hip arthroplasty (THA). While the procedure is well established as an effective treatment for relieving pain and restoring function in degenerative as well as traumatic hip conditions, the initial recovery period is frequently marked by balance difficulties, restricted mobility and heightened concern about falling [1]. Previous reports suggest that fall incidence is highest within the first postoperative year, with almost one-third of patients experiencing at least one fall [2, 3]. Such episodes carry consequences beyond the immediate risk of injury, often resulting in loss of independence, psychological distress, diminished quality of life and increased health care utilisation [4]. A variety of factors have been implicated in post-THA falls. Weakness of the hip abductors, particularly in patients with a prior history of falling, has been consistently identified as a prominent risk factor [2]. In addition, declines in muscle strength and balance associated with ageing, comorbid conditions such as diabetes or hypertension and reduced confidence in completing everyday tasks further increase susceptibility [5]. The psychological dimension is also important: fear of falling-commonly assessed using the Falls Efficacy Scale (FES)-tends to be elevated in this group [6, 7]. Higher FES scores, which indicate lower confidence, have frequently been associated with limitations in mobility and functional activity [8]. Because of these risks, structured monitoring of recovery is vital for detecting patients who may be vulnerable to falls. Simple but validated clinical measures such as the Timed Up and Go (TUG), Sit-to-Stand (STS) and Alternate Step Test (AST) are widely applied in arthroplasty and geriatric rehabilitation research [9, 10]. These assessments provide practical insight into balance, coordination, strength and mobility-factors fundamental to safe walking. Their predictive value for falls has been demonstrated in several studies, reinforcing their relevance in routine postoperative follow-up [11, 12]. Patient-reported and clinician-administered outcome measures also contribute important perspectives to recovery assessment. The Harris Hip Score (HHS) continues to be one of the most frequently used tools for evaluating pain, functional ability and deformity after hip replacement [13]. Although ceiling effects have been noted in some populations, the scale remains valuable in the early postoperative phase. When combined with the FES and functional test outcomes, it enables a more comprehensive appraisal of rehabilitation progress [14]. Current evidence indicates that both physical impairments and psychosocial factors influence fall risk before and after THA [15, 16]. Appreciating how physical recovery, fall risk and confidence interact highlights the importance of prospective research that considers these factors together. Such studies can guide the design of rehabilitation strategies that not only improve functional capacity but also enhance patient confidence and reduce fall risk. Therefore, it is of interest to determine the changes in fall risk and functional recovery during the first three months after THA in older adults. Specifically, the study aimed to track recovery patterns using HHS, FES, TUG, STS and AST and to explore the associations between fall-related confidence and functional performance.

## Materials and Methods:

The study was carried out on older adults who had undergone total hip arthroplasty (THA). Men and women aged 60 years or more were taken as participants. Their first intital assessment for the study was done around 3 to 4 weeks after surgery.

Inclusion criteria were preserved cognitive function (Mini-Mental State Examination score >26) and Provision of written informed consent.

Exclusion criteria were femoral shaft or acetabular fractures treated with fixation devices (e.g., Dynamic Hip Screw, Proximal Femoral Nail), Partial hip replacement, Active hip infection, including tuberculosis. A total of 43 patients who matched the study criteria were included and they were observed throughout the course of the study.

## Patients were evaluated at three postoperative intervals:

[1] 3-4 weeks

[2] 6-7 weeks

[3] 12-13 weeks

[4] At each follow-up, fall-related outcomes and hip function were assessed using standardized measures.

[5] Functional testing was conducted only after receiving medical clearance from the treating orthopedic surgeon.

[6] All assessments were performed in a controlled and safe clinical setting.

## Outcome Measures calculate are:

[1] Harris Hip Score (HHS): Evaluates pain, function, deformity and range of motion in the hip joint.

[2] Falls Efficacy Scale (FES): Measures self-perceived confidence in carrying out daily activities without falling.

[3] Timed Up and Go (TUG): Records the time required to rise from a chair walk three meters, turn, return and sit.

[4] Sit-to-Stand Test (STS): Assesses lower-limb strength and balance through both single and five-repetition formats.

[5] Alternate Step Test (AST): Evaluates balance and coordination by alternating steps onto a raised platform.

Data were analyzed using SPSS software. Changes across the three assessment periods were examined with Friedman's test for repeated measures. Relationships between fall efficacy and functional test performance at 12-13 weeks were analyzed using Spearman's correlation coefficient. A p-value less than 0.05 were considered statistically significant.

## Results:

A total of 43 geriatric patients (29 males, 14 females) participated. The mean age was 69.7 ± 10.0 years and the mean body mass index (BMI) was 25.7 ± 1.8 (Table 1 - see PDF). The most common indication for THA was femoral neck fracture (53.4%), followed by avascular necrosis (27.9%) and osteoarthritis (18.7%) (Table 2 - see PDF). Regarding comorbidities, hypertension was observed in 18.6%, diabetes mellitus in 20.9% and both conditions in 16.3% of the sample (Table 3 - see PDF).

Friedman's test revealed statistically significant improvements across all variables (p < 0.001) (Table 4 - see PDF, 5 - see PDF).

[1] HHS: Increased from 61.3 at 3-4 weeks to 68.3 at 12-13 weeks.

[2] FES: Declined from 71.4 to 63.8, reflecting reduced fear of falling.

[3] STS: Improved from 35.6 seconds to 29.6 seconds.

[4] AST: Improved from 28.2 to 23.1 seconds.

[5] TUG: Improved from 55.2 to 46.8 seconds.

At 12-13 weeks, fear of falling (FES) was most strongly correlated with Sit-to-Stand (r = 0.383, p = 0.011), moderately correlated with AST (r = 0.315, p = 0.040) and weakly correlated with TUG (r = 0.300, p = 0.051) (Table 6 - see PDF). Figure 1 (see PDF) shows the change in Harris Hip Score (HHS) and Falls Efficacy Scale (FES) across three postoperative intervals (3-4 weeks, 6-7 weeks, 12-13 weeks). Figure 2 (see PDF) shows improvement in Sit-to-Stand (STS) test times across the three postoperative intervals. Figure 3 (see PDF) shows improvement in Alternate Step Test (AST) times across the three postoperative intervals. Figure 4 (see PDF) shows the improvement in Timed Up and Go (TUG) test times across the three postoperative intervals. Figure 5 (see PDF) shows the correlation between Falls Efficacy Scale (FES) scores and Sit-to-Stand (STS) performance at 12-13 weeks (r = 0.383, p =0.011).

## Discussion:

The present study demonstrated progressive improvements in hip function, mobility and fall-related confidence during the first three months after THA. Patients showed measurable gains across all assessment tools, with both clinical scores (HHS, FES) and functional performance tests (STS, AST, TUG) reflecting steady recovery. These findings align with prior work reporting marked postoperative improvements in pain relief, mobility and overall function [13, 14]. The consistent rise in HHS underscores the early effectiveness of THA in restoring hip function. While long-term ceiling effects are known to limit the HHS [13], its responsiveness in the short term supports its utility for monitoring early outcomes. One of the most encouraging results was the decline in FES scores, suggesting reduced anxiety and greater confidence in daily activities. Fear of falling is widely recognized as a barrier to independence in older adults [3, 6]. Previous studies have noted that such fear may persist despite functional gains [7, 11], but in this cohort confidence improved alongside mobility, reflecting positive psychological adaptation. Marked improvements in STS, AST and TUG highlight recovery of balance, coordination and lower-limb strength, all of which are critical for reducing fall risk [9, 10]. The strong correlation between FES and STS emphasizes the role of leg strength in shaping self-efficacy, echoing evidence that hip muscle weakness is a major risk factor for falls [2, 16]. The moderate association with AST further supports its role in assessing dynamic balance. In contrast, the weaker correlation between FES and TUG suggests that while TUG is a useful general mobility measure, it may not fully capture the strength and balance dimensions that underpin fall confidence. The recovery trajectory observed here mirrors earlier reports that falls are most common in the first postoperative months and decline thereafter [2, 3]. Importantly, improved mobility does not always translate into fewer falls; some patients continue to experience falls in everyday situations despite functional recovery [15]. This highlights the complex interplay between physical capacity, behavior and environment. Our findings support a multidimensional approach to monitoring recovery after THA. Using both patient-reported (FES, HHS) and performance-based (STS, AST, TUG) tools provides a more complete picture than relying on a single measure. Identifying patients with persistent fear of falling despite good physical recovery can help target them for additional interventions such as balance training, strength programs, or psychological support. Early attention to hip abductor strengthening may also be valuable in reducing fall vulnerability [16, 17].

## Conclusion:

The study found significant improvements in hip function, strength, balance and mobility in older adults after total hip arthroplasty. Additionally, a reduction in fall fear highlighted increased confidence in daily tasks. Functional tests like Sit-to-Stand and Alternate Step Test are valuable tools for tracking recovery and identifying fall risk.
